# HUMAN RESOURCES PROFESSIONALS’ PERCEPTIONS OF AGE-FRIENDLY EMPLOYMENT

**DOI:** 10.1093/geroni/igz038.2955

**Published:** 2019-11-08

**Authors:** Mi Sun Choi, Holly Dabelko-Schoeny, Katie White, Marisa Sheldon

**Affiliations:** The Ohio State University, Columbus, Ohio, United States

## Abstract

Many researchers have attempted to find alternative ways to address the challenges of the aging workforce. Applying the concept of age-friendly to work settings may be a promising strategy to promote older adults’ well-being in the workplace. However, age-friendly employment is an abstract concept, and empirical research identifying its features is lacking. Also, few studies adequately represented the diverse characteristics of different industries and stakeholder’s perceptions of age-friendly work environments. This study used focus group data and the Delphi method, along with existing literature to create an instrument to measure age-friendliness in diverse work environments. In partnership with a senior employment organization serving central Ohio, six human resources (HR) professionals participated in a focus group discussion and 10 HR professionals participated in a two-round Delphi study. In the focus group discussion, HR professionals identified flexibility, mobility, ergonomics, health benefits, and respect as age-friendly aspects in the workplace. We developed 37 items within five subcategories: accommodation, development, maintenance, utilization, and inclusion. In the first round, HR professionals assessed the importance of each item, presenting training opportunities for employees of all ages as the most important practice; four weeks later they examined the revised 33 items in regard to the feasibility of their real-world implementation, showing 100% agreements among experts in career advice, opportunities for promotion, involving decision making, educating age discrimination, and formal acknowledgment. The second phase of this study will include scale evaluation to confirm its reliability and validity with a broader number of HR professionals and older adults.

